# Nutritional deficiency in South African adults scheduled for bariatric surgery

**DOI:** 10.3389/fendo.2023.1120531

**Published:** 2023-05-12

**Authors:** Prabash Sadhai, Ankia Coetzee, Marli Conradie-Smit, C. J. Greyling, Rutger van Gruting, Inge du Toit, Jeanne Lubbe, Mari van de Vyver, Magda Conradie

**Affiliations:** ^1^ Tygerberg Hospital, Faculty of Medicine and Health Sciences, Stellenbosch University, Cape Town, South Africa; ^2^ Division of Endocrinology, Department of Medicine, Faculty of Medicine and Health Sciences, Stellenbosch University, Cape Town, South Africa; ^3^ Specialist Physician & Endocrinologist, Durbanville Mediclinic and Kuilsriver Netcare Hospital, Cape Town, South Africa; ^4^ Department of Surgical Sciences, Division of Surgery, Faculty of Medicine and Health Sciences, Stellenbosch University, Cape Town, South Africa; ^5^ Division of Clinical Pharmacology, Department of Medicine, Faculty of Medicine and Health Sciences, Stellenbosch University, Cape Town, South Africa

**Keywords:** bariatric (weight loss) surgery, obesity, micronutrient deficiency, pre-bariatric aspects, diabetes, diabetes mellitus, vitamin D

## Abstract

**Background:**

Globally, there is a rising trend in obesity, known to increase morbidity and mortality. Metabolic surgery and adequate weight loss decrease mortality but may worsen pre-existing nutrient deficiencies. Most data on pre-existing nutritional deficiencies in the population undergoing metabolic surgery is from the developed world, where an extensive micronutrient assessment is achievable. In resource-constrained environments, the cost of a comprehensive micronutrient assessment must be weighed against the prevalence of nutritional deficiencies and the potential harm if one or more nutritional deficiencies are missed.

**Methods:**

This cross-sectional study investigated the prevalence of micronutrient and vitamin deficiencies in participants scheduled to undergo metabolic surgery in Cape Town, South Africa, a low-middle income country. 157 participants were selected and 154 reported on; who underwent a baseline evaluation from 12 July 2017 to 19 July 2020. Laboratory measurements were conducted, including vitamin B12 (Vit B12), 25-hydroxy vitamin D (25(OH)D), folate, parathyroid hormone (PTH), thyroid-stimulating hormone (TSH), thyroxine (T4), ferritin, glycated haemoglobin (HbA1c), magnesium, phosphate, albumin, iron, and calcium.

**Results:**

Participants were predominantly female, aged 45 years (37-51), with a preoperative BMI of 50.4 kg/m^2^ (44.6-56.5). A total of 64 individuals had Type 2 diabetes mellitus (T2D), with 28 undiagnosed cases at study entry (18% of study population). 25(OH)D deficiency was most prevalent (57%), followed by iron deficiency (44%), and folate deficiency (18%). Other deficiencies (vitamin B12, calcium, magnesium, phosphate) were rarely encountered and affected ≤1% of participants. Folate and 25(OH)D deficiency were related to obesity classification, with a higher prevalence in participants with a BMI ≥40 kg/m^2^ (p <0.01).

**Conclusion:**

A higher prevalence of some micronutrient deficiencies was noted compared with data from similar populations in the developed world. The minimum baseline/preoperative nutrient evaluation in such populations should include 25(OH)D, iron studies, and folate. Additionally, screening for T2D is recommended. Future efforts should seek to collate broader patient data on a national scale and include longitudinal surveillance after surgery. This may provide a more holistic picture of the relationship between obesity, metabolic surgery and micronutrient status inform more appropriate evidence-based care.

## 1 Introduction

The unfavorable metabolic milieu associated with obesity is a worldwide concern and was recently highlighted by the Coronavirus Infectious Disease 2019 (COVID-19) pandemic (1–4). Epidemiological studies from around the globe have conclusively demonstrated a linear association between obesity and all-cause mortality, with mortality pre-dominantly attributable to cardiovascular disease (1, 5, 6). A recent meta-analysis suggests a rise in death rate as body mass index (BMI) increases above 25 kg/m^2^ (7).

Obesity is often mistakenly assumed to be synonymous with a state of nutritional excess. The excess, however, only applies to the energy balance. As such obesity can paradoxically represent a state of nutritional deficiency. Obesity is often compounded by a change in diet to low-cost, readily available, high sugar, high carbohydrate, and nutrient-deficient meals. Therefore, excess weight can co-occur with malnutrition, known as the ‘overfed but undernourished’ phenomenon (8, 9).

Metabolic surgery effectively addresses the harmful effects of excess weight and has decreased morbidity and mortality associated with excess adiposity (10–13). The benefits of metabolic surgery performed in high volume centers outweigh the associated potential harm, provided that precautionary measures are taken to prevent long-term adversities (14–17).

In recent years reports of pre-existing micronutrient deficiency in patients scheduled for metabolic surgery have increased (18, 19). Krzizek et al. (19) demonstrated a high prevalence of micronutrient deficiencies preoperatively in Austria, with 25-hydroxy vitamin D (25(OH)D) deficiency significantly associated with higher BMI (19). Flancbaum reported preoperative deficiencies of 43.9% for iron, 8.4% for ferritin, and 68.1% for 25(OH)D in a cohort from New York (18). Geographical, socio-economical, and ethnic variables limit extrapolation of these findings to the South-African population undergoing surgery for obesity.

Since metabolic surgical procedures may further impact micronutrient status, correcting pre-existing deficiencies is standard care. It is thus recommended to evaluate and optimize the micronutrient status of all patients scheduled to undergo metabolic surgery preoperatively (20–22). In resource-limited environments, the cost of a comprehensive preoperative micronutrient assessment needs to be weighed against both the prevalence of deficiencies and the potential harm if these deficiencies remain undetected. Recommendations on preoperative testing should ideally be contextualized. The primary objective of this study was to determine the prevalence of micronutrient and vitamin deficiencies in obese patients scheduled to undergo metabolic surgery at a tertiary referral centre from a low-middle income country.

## 2 Materials and methods

This study, nested within a larger prospective OMIT (Obesity and Metabolic surgery Initiative Tygerberg) cohort study, followed a descriptive, cross-sectional design. The OMIT study was designed to evaluate the safety and efficacy of high-volume metabolic surgery in the public health sector at Tygerberg Hospital. Tygerberg Hospital is a tertiary level hospital that serves as a referral center for more than 3.4 million people residing in the Cape Town Metropole of South Africa.

The study protocol was approved by Stellenbosch University’s Health Research Ethics Committee (S18/01/003) and conducted according to the 1964 Helsinki Declaration principles. All participants provided written informed consent.

### 2.1 Inclusion and exclusion criteria

All patients scheduled to undergo a Roux and Y-gastric bypass procedure or sleeve gastrectomy from 12 July 2017 to 19 July 2020, who underwent a thorough preoperative assessment, were considered for inclusion in this analysis.

#### 2.1.1 Inclusion criteria

Obesity was defined based on the World Health Organization (WHO) classification (23). Obese individuals, aged 20-60-years, were eligible for surgical intervention and inclusion in the OMIT study, if they met the following criteria:

Individuals with

o BMI ≥40 kg/m^2^ (WHO class III and above)
o BMI 30.0-39.9 kg/m^2^ (WHO class I and II) with a related co-morbid disease (diabetes, obstructive sleep apnea, osteoarthritis etc.), prior gestational diabetes (GDM) or current pre-diabetes (i.e., high risk for future diabetes)

#### 2.1.2 Exclusion criteria

Potential participants were discussed by a multidisciplinary team (MDT) and were excluded from study entry if any of the following conditions were noted:

o uncontrolled medical comorbidity(ies)
o concurrent comorbidity(ies) deemed to be too high an operative risk following evaluation by a specialist anesthetist
o contraindications to laparoscopic metabolic surgery including multiple prior open abdominal surgeries and the lack of commitment, support, resources, or other significant barriers to short, medium, and long-term adherence to the program.
o active underlying malignancy or bowel disease such as peptic ulcer disease or inflammatory bowel disease
o uncontrolled psychiatric illness or unwillingness to undergo psychiatric evaluation towards assessing psychological “fitness” for metabolic surgery
o excessive, current, or previous alcohol or drug dependence/abuse
o current pregnancy, breastfeeding, planning pregnancy in the next two years or unreliable contraception
o immobile patients

### 2.2 Sociodemographic and anthropometric measurements

Data was collected prospectively. Gender and ethnic determination were made by self-declaration. Socio-economic status was categorized using the South African Department of Health’s Uniform Patient Fee Schedule’s (UPFS) classification code. The classification code, usually expressed in South African Rands, is reflected here as US Dollars for ease of reference. It is based on annual income brackets as follows: H0: Low – receiving grant or pension; H1: <$3800/person or <$5450/family; H2: $3800-$5450/person or $5450-$19070/family; H3: >$13620/person or >$19070/family; P: Private patients are those not subsidized by the state and, for this research, denotes participants with medical aid or the highest income category.

Basic anthropometric measurements (weight, height, waist circumference) were obtained by dedicated and permanent nursing staff. Weight was measured with minimal clothing using a Charder^®^ digital scale placed on a flat surface and recorded to the nearest kilogram (kg) and height to the nearest one centimeter (cm) with a calibrated stadiometer. Body mass index (kg/m^2^) was calculated as weight (kg) divided by square height (m) and participants were categorized according to the WHO classification (23): overweight (25-29.9 kg/m^2^); Class I obesity (30-34.9 kg/m^2^); Class II obesity (35-39.9 kg/m^2^); Class III obesity (>40 kg/m^2^) (23). Waist circumference (WC) was measured to the nearest 0.5 cm with a non-stretchable tape measure.

The clinical assessment also included blood pressure, urine dipstick, and a thorough surgical risk evaluation. Brachial blood pressure (BP) was measured, once, on the left arm while sitting down using a calibrated automated sphygmomanometer (Dinamap Carescape V100) with an appropriate cuff size. RightSign^®^ multistix were used for urine analysis.

### 2.3 Biochemical analysis

All laboratory measurements were done by the National Health Laboratory Service (NHLS), a SANAS (South African National Accreditation System) accredited laboratory at Tygerberg Hospital (accreditation code M0390) using the Roche Cobas®6000 analyzer. The NHLS adheres to the Clinical and Laboratory Standards Institute (CLSI, previously National Committee for Clinical Laboratory Standards [NCCLS]) EP28-A3c (formerly C28-A3) guideline for Defining, Establishing and Verifying Reference Intervals in the Clinical Laboratory (24). Reference intervals used in our study are not population specific, but provided by the instrument manufacturer (24).

#### 2.3.1 Micronutrient and hormonal assessment

Micronutrient status and associated hormonal regulators were determined *via* various laboratory techniques that included the following: electrochemiluminescence binding assay (immunoassay) for Vitamin B12 (Vit B12), 25(OH)D, folate, parathyroid hormone (PTH), thyroid-stimulating hormone (TSH), thyroxine (T4), and ferritin measurements; turbidimetric inhibition immunoassay for glycated hemoglobin (HbA1c); immunoturbidimetric assay to measure transferrin; colorimetric assay for magnesium, phosphate, albumin, and iron and spectrophotometric detection for calcium. Serum copper, zinc, selenium, vitamins B1, B3, B6, A, E, K, and C were not assessed. The HbA1c percentage was calculated with a programmed equation adhering to the international federation of clinical chemistry and laboratory standard recommendations (25).

Deficiencies were defined as follows: albumin <35 g/L, vitamin B12 <145 pmol/L, calcium <2.15 mmol/L, 25(OH)D deficient if ≤50 nmol/L; insufficient if 50.1-72.5 nmol/L and sufficient if ≥72.5 nmol/L (26); iron ≤13 ug/L, serum folate <8.8 nmol/L, magnesium <0.63 mmol/L, phosphate <0.78 mmol/L. Iron deficiency was defined as a ferritin <30 mcg/l or ferritin <100 mcg/l plus transferrin saturation <20 percent. Iron deficiency anemia was defined as a Hb <13 g/dl in male participants and <12 g/dl in female participants with biochemical features of iron deficiency (25).

Hormonal assessments were further defined or categorized. If the TSH was outside of the laboratory reference range (0.27–4.2 mIU/L), a free T4 and T3 were measured. Patients with a raised (PTH >6.9 pmol/L) or inappropriately normal PTH level (3.6-6.9 pmol/L) were diagnosed as having primary hyperparathyroidism if calcium was elevated (> 2.51 mmol/L). If calcium was not elevated, secondary hyperparathyroidism applied if 25(OH)D levels was noted to be deficient. Normocalcemic hyperparathyroidism was diagnosed if calcium and 25(OH)D were normal with a concomitant PTH >6.9 pmol/L.

#### 2.3.2 Assessment of glycemic status

Glycemic status was not formally assessed with a 75-gram glucose 2-hour oral glucose tolerance test (OGTT). A diagnosis of pre-diabetes was based on a fasting plasma glucose (FPG) ≥5.6 mmol/L and <7 mmol/L and/or a HbA1c ≥5.7% and <6.5% (≥39 mmol/mol and <48 mmol/mol) (27). Diabetes mellitus was defined as a participant known with diabetes mellitus on anti-diabetic medication, or patients not known with diabetes mellitus with a FPG ≥7 mmol/L and/or HbA1c ≥6.5% (≥48 mmol/mol) (27).

### 2.4 Data management and statistical analysis

Structured standardized data sheets were used to capture demographic characteristics and relevant clinical data and subsequently gathered in the Bariatric Outcomes Longitudinal Database (BOLD), managed by the Surgical Review Corporation (SRC). The SRC, founded in 2003, is a United States of America-based independent and non-profit organization offering accreditation and assistance with data management. BOLD is web-based, and data was entered at each encounter or during bulk transfer from clinical notes. Statistical analysis was performed using GraphPad Prism (version 9) software. The Kolmogorov-Smirnov (K-S) normality test with Lilliefors correction was used (p<0.05) and normal distribution of data confirmed with the Shapiro-Wilk test. Non-parametric data is presented as median (IQR). Chi-square analysis and Fisher’s exact tests were used to determine differences between stratified categories. Kruskal-Wallis ANOVA with Dunn’s multiple comparison or Mann-Whitney test was done to determine differences between groups. Spearman’s simple linear regression analysis was used to determine associations between variables. A probability value (p-value) of <0.05 was regarded as significant.

## 3 Results

### 3.1 Sociodemographic and anthropometric assessments

**Figure 1:**
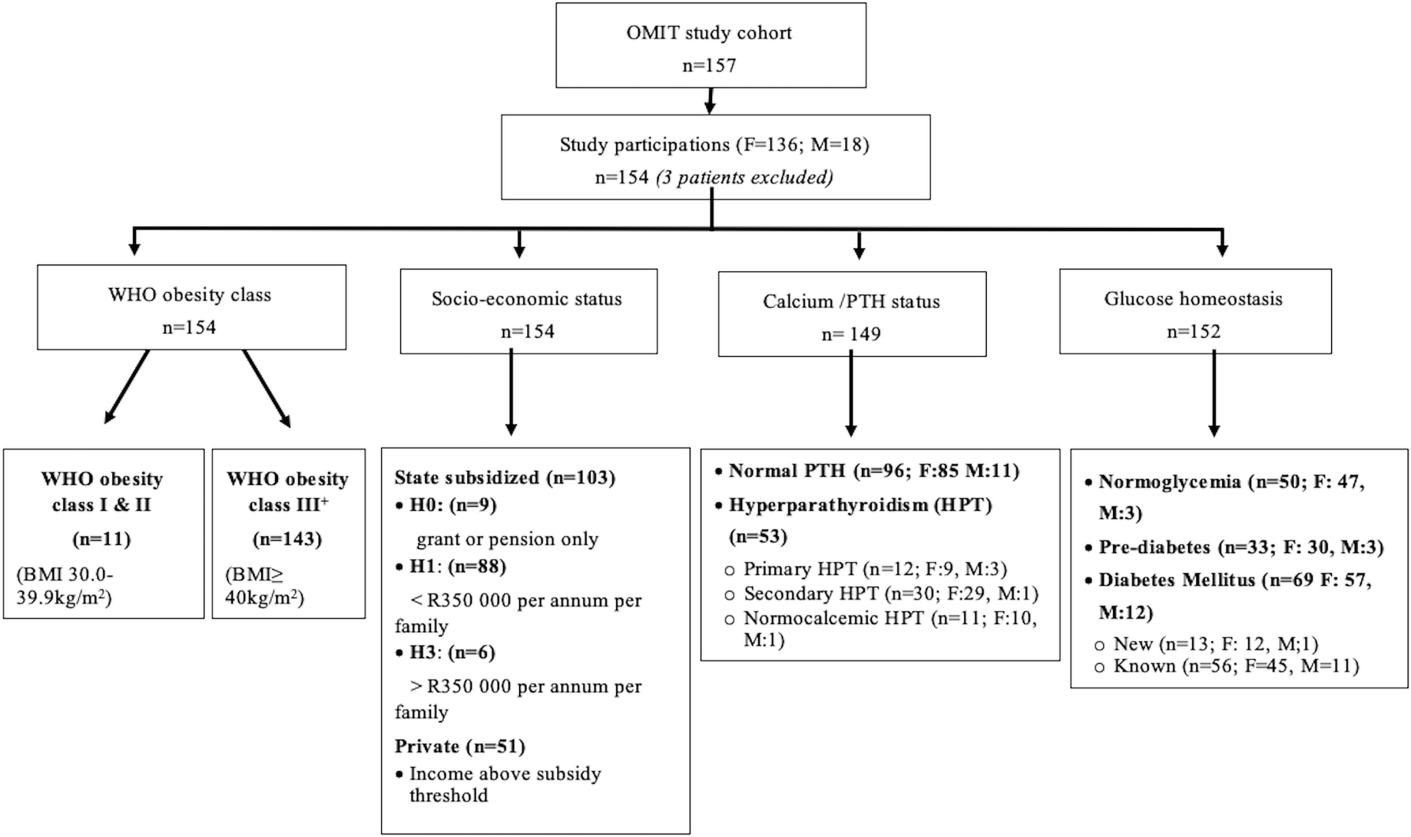
Overview of the study population and sstratification categories based on glycemic status and calcium homeostasis.

Of the 157 participants referred for metabolic surgery in the 3-year study-period, 154 (98%) were eligible for inclusion (**Figure 1**). Eleven participants (7%) were diagnosed with Class I or II obesity (<40 kg/m^2^), while all others presented with Class III obesity or higher (>40 kg/m^2^) (**Supplementary Table S1**). The median BMI of the total cohort was above the WHO Class III threshold [50.4 kg/m^2^ (44.6-56.5)]. Most participants (136/154) were female with a median age of 45 years (37–51) and the majority either of mixed or Asian (65/154; 42%) or European (83/154; 54%) descent. Three patients were excluded due to concurrent comorbidity (significant chronic renal failure) deemed too high perioperative risk. Amongst the public-sector participants (n=103; 66% of study cohort), annual income in most participants was between $3800 and $5450 as individual and/or between $5450 and $19070 per family (categories H1-2) in 88/103 (85%). A small minority of public sector participants relied on state pension or medical disability (n=9; 9%; H0) or had a personal or family income more than $13620 or $19070 respectively (n=6; 6%; H3). Non-subsidized private participants on medical insurance comprised 33% of the study cohort.

### 3.2 Baseline micronutrient deficiencies and hormonal assessment

**Figure 2:**
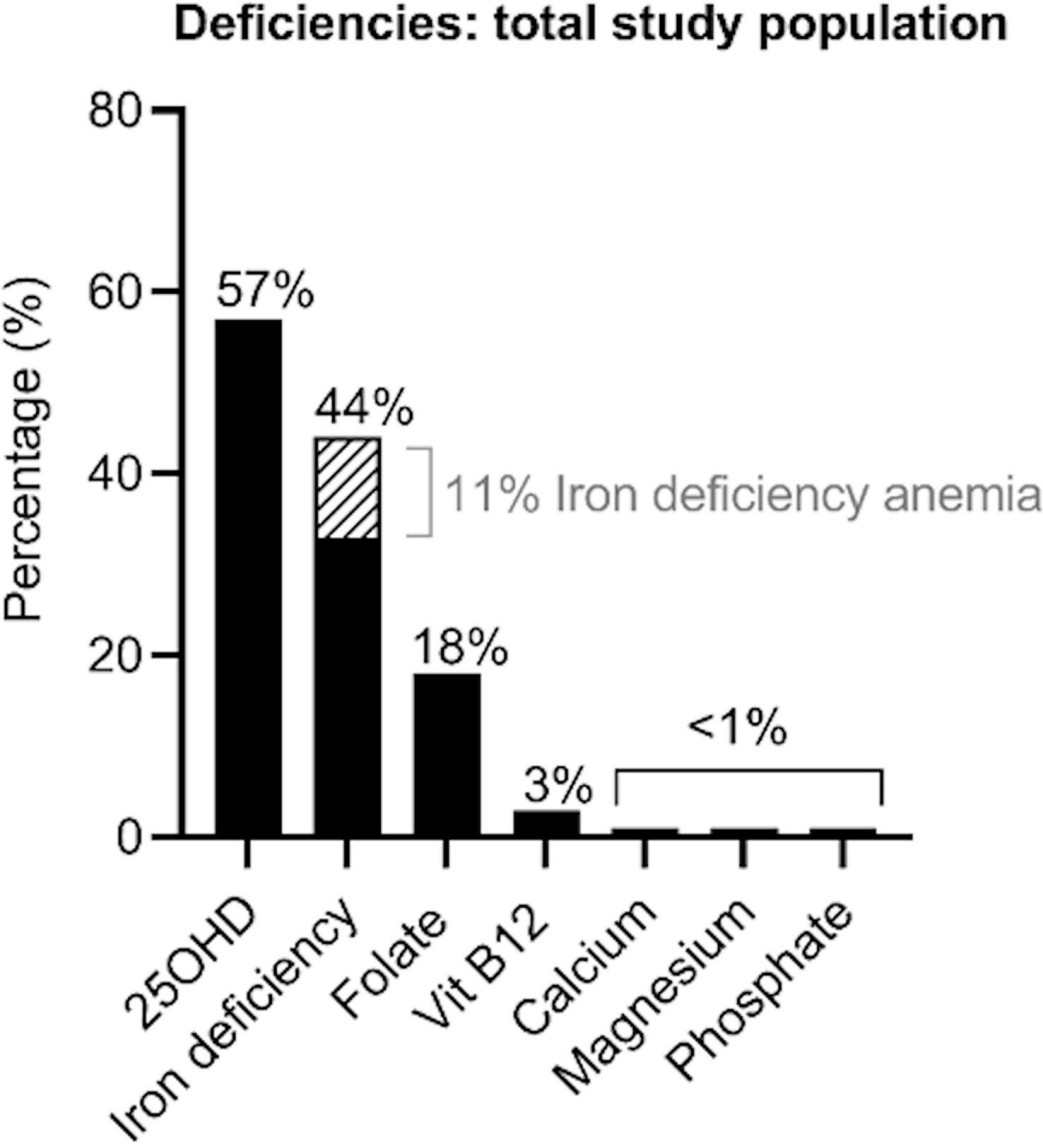
Overview of the deficiencies evident in the total study population.

An overview of the micro-nutrient deficiencies in the study population at baseline is presented in **Figure 2**. The most common deficiency amongst the study population was a 25(OH)D deficiency, noted in 57% (88/154) of the cohort. Iron deficiency was present in 44% of participants, and folate deficiency in 18%. Deficiency of vitamin B12 (3%), calcium (1%), magnesium (1%) and phosphate (1%) were limited. None of the participants with vitamin B12 deficiency was on chronic metformin treatment and only one of the five was on a proton pump inhibitor (PPI). A total of 124 (80%) participants had 1-2 micronutrient deficiencies, 12 had >2 deficiencies and 18 did not have any deficiencies.

Baseline micronutrient deficiencies (**Table 1**) and diabetes status (**Supplementary Table S1**) were tabulated within population subcategories based on ethnicity, annual income and obesity class. Statistical evaluation was limited to ethnicity and obesity class on 25(OH)D deficiency due to too small numbers for the other micronutrient deficiencies within the mentioned subpopulations.

**Table 1:** Baseline micronutrient deficiencies in the total cohort and within certain population categories.

	25(OH)D *(<50 nmol/L)*	Calcium *(<2,15 mmol/L)*	Iron *(<13 ug/L)*	Ferritin *(<30mcg/dL)*	Transferrin Sats (<20%)	Haemoglobin (<12 g/dL)	Albumin *(<35g/L)*	Iron deficiency^1^	Iron deficiency Anemia^2^	Folate *(<8,8 nmol/L)*
Total study population: N=154										
* median (IQR)*	46 (37-62)	2.38 (2.3-2.47)	10.95 (8.2-13.6)	76 (38-170)	16 (12-20)	13.5 (12.5-14.3)	44 (42-46)			17.0 (10.3-28.4)
Total number of deficiencies	88/154 (57%)	2/154 (1%)						66/150 (44%)	17/150 (11%)	27/143 (18%)
Number of deficiencies per category:										
Gender										
Male	9/18 (50%)	0	8/18 (44%)	0	11/18 (61%)	0	0	3/18 (17%)	1/18 (5%)	3/18 (17%)
Female	79/136 (58%)	2/136 (1%)	105/136 (77%)	21/136 (15%)	107/136 (78%)	17/136 (12%)	0	63/136 (46%)	16/136 (12%)	24/136 (18%)
Ethnicity										
Mixed or Asian	51/65 (78%)	1/65 (2%)	52/65 (80%)	13/65 (20%)	53/62 (85%)	10/63 (16%)	0	34/65 (52%)	8/65 (12%)	10/62 (16%)
European	33/83 (40%)	1/83 (1%)	51/83 (61%)	7/82 (9%)	51/80 (64%)	6/83 (7%)	0	32/83 (39%)	8/83 (10%)	17/79 (22%)
Black African	4/6 (67%)	0	3/6 (50%)	1/6 (17%)	3/6 (50%)	1/6 (17%)	0	0	1/6 (17%)	0
* Chi-square*	*p<0.01									
Annual Income										
H0	4/9 (44%)	0	7/9 (78%)	1/9 (%)	7/9 (78%)	1/9 (11%)	0	5/9 (56%)	1/9 (11%)	0
H1-2	53/88 (60%)	1/88 (1%)	62/88 (70%)	12/88 (14%)	61/88 (69%)	7/88 (8%)	0	36/88 (41%)	6/88 (7%)	19/88 (22%)
H3	1/6 (17%)	0/6 (0%)	3/6 (50%)	0	3/6 (50%)	0	0	4/6 (67%)	0	1/6 (17%)
P	30/51 (59%)	1/51 (2%)	34/49 (69%)	7/50 (%)	36/45 (80%)	9/48 (19%)	0	21/49 (43%)	2/49 (4%)	6/46 (13%)
* Chi-square*	–									
Obesity Class										
Class I-II BMI<40kg/m^2^	3/11 (27%)	0						2/11 (18%)	1/11 (9%)	0
Class III or above BMI>40kg/m^2^	85/143 (59%)	2/143 (1%)						64/143 (45%)	16/143 (11%)	27/143 (19%)
* Fishers Exact*	*p<0.01									

Statistical analysis, Fishers Exact & Chi-square analysis. *p<0.05 indicate significance.

**Figure 3:**
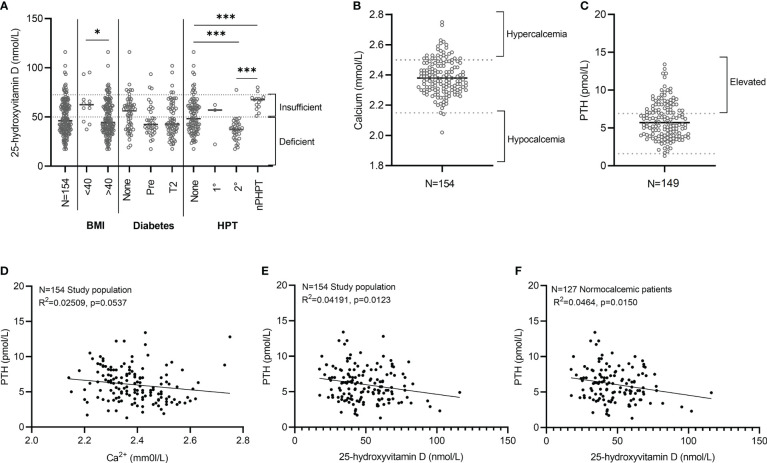
Relationship between PTH and 25(OH)D. **(A)** 25-hydroxyvitamin D (nmol/L). Individual data points are presented with the median for each group indicated using a grey line. The number of patients per category is as follow: BMI<40 kg/m^2^ (N=11), BMI>40kg/m^2^ (N=143), No diabetes (N=54), Pre-diabetes (N=34), Type 2 diabetes (N=64), Normal PTH levels (N=98), Primary hyperparathyroidism (HPT) (N=3), Secondary HPT (N=34), Normocalcemic HPT (N=14). *Statistical analysis.* BMI category: Mann-Whitney test. Hyperparathyroidism category: Kruskal-Wallis multiple comparisons test. *p<0.05, ***p<0.001 indicate significant difference between groups **(A)**. **(B)** Calcium levels (mmol/L) (N=154). **(C)** PTH levels (pmol/L) (N=154). Individual values are presented for the total study population (N=154) with the median indicated using a black line **(B, C)**. **(D–F)** Simple linear regression analysis between PTH and Calcium **(D)** and 25-hydroxyvitamin D **(E, F)**.

A significant association between 25(OH)D deficiency (≤50 nmol/L) and ethnicity was noted in the study cohort (**Table 1**). 25(OH)D deficiency was prevalent in 78% (51/65) (n/N) of participants with Mixed/Asian ancestry, in 67% (4/6) (n/N) of participants with Black African ancestry, and only in 40% (33/83) (n/N) of participants with European ancestry. There was furthermore a significant difference in the median 25(OH)D levels amongst WHO obesity categories. Class I and II obese participants (BMI<40kg.m^2^) had higher 25(OH)D levels [62 (45–67) nmol/L] compared to class III obese participants (BMI>40 kg.m^2^) [44 (36–60) nmol/L]. The majority (85/143; 59%) of individuals with BMI ≥40 kg/m^2^ had 25(OH)D deficiency, compared to 27% (3/11) with a BMI <40 kg/m^2^ (**Figure 3A** and **Table 1**).

Circulating plasma calcium levels were available in 154 participants and PTH status documented in 149. Calcium levels were low, normal, and elevated in 1% (2/154), 82% (127/154) and 16% (25/154) of patients at baseline respectively (**Figure 3B**). The two participants with low calcium levels had mild and asymptomatic hypocalcemia (i.e., uncorrected calcium levels of 2.02 mmol/L (albumin 42 g/dL) and 2.14 mmol/L (albumin 44 g/dL). A complete calcium and PTH dataset were available in 149 participants that included 17 of the 25 participants with hypercalcemia (**Figure 3C**). The diagnosis of primary hyperparathyroidism was based on the presence of hypercalcemia with either concomitant elevated PTH levels (4/12) or inappropriately normal, unsuppressed PTH values i.e., a PTH >3.5-6.9mmol/L (8/17). Primary hyperparathyroidism was present in 8% (12/149) [12/17 with hypercalcemia], secondary hyperparathyroidism in 20% (30/149), and normocalcemic hyperparathyroidism in 7% (11/149).

An expected inverse relationship between PTH and both calcium and 25(OH)D was observed (**Figures 3D, E**). The inverse relationship between PTH and 25(OH)D remained significant in the presence of normal circulating calcium levels (**Figure 3F**). Thirty four of the 88 participants with deficient 25 (OH)D levels had physiologically appropriate secondary hyperparathyroidism. WHO obesity class and glucose status had no statistically significant impact on circulating calcium levels.

Iron deficiency was the second most prevalent deficiency. Sixty-six participants (66/150; 44%) met the criteria for iron deficiency and 17/150 (11%) had iron-deficiency anaemia (**Table 1**). Folate deficiency was not present in any of participants in the BMI <40 kg/m^2^ (0/11) (n/N) whereas 18% of participants with BMI ≥40 kg/m^2^ (27/143) (n/N) presented with serum folate ≤8.8 nmol/L.

The median TSH of the total cohort was 1.97 (1.42-2.99) mIU/L. Of the 18 participants who had an elevated TSH level at baseline, the majority were known with primary hypothyroidism (10/18) on levothyroxine replacement therapy and of them 14 had subclinical hypothyroidism only (normal T4, TSH 4.2-9.9 mIU/L).

### 3.3 Glycemic status

A complete data set to evaluate glycemic status was available for 152 of the study participants (**Figure 1**). A concerning percentage of the cohort had abnormal glucose homeostasis at baseline (n=102; 67%). T2D was present in 45% and included 69 participants, 56 with previously known and 13 with newly diagnosed T2D. The mean HbA1c in those with known T2D on treatment was 7.4 ± 1.8% and 7.3 ± 1.9% in those with newly diagnosed T2D prior to any intervention. Optimal glycemic control (treatment target HbA1c ≤ 7%) was only evident in 24 of the 56 participants with known T2D (42.8%). Pharmacological therapy in most was limited to monotherapy with metformin (n=40/56; 71%). Fourteen participants used combination oral therapy (metformin ± another oral agent) with or without insulin. Two participants were on insulin only.

## 4 Discussion

This study is the first to define preoperative micronutrient status in an African patient cohort scheduled for metabolic surgery. In South Africa, literature is available on the baseline patient profiles and outcomes after metabolic surgery, but little is known about the preoperative micronutrient status of bariatric cohorts (28). This study, in predominantly morbidly obese females, revealed significant deficiencies of some micronutrients at baseline indicative of obesity as a state of excess energy and is not synonymous with optimal nutrition. Like data from the developed world, vitamin D deficiency (57%) was the most prevalent, followed by iron deficiency (44%) and then folate deficiency (18%); with 88% (136/154) of the participants having at least one nutrient deficiency prior to surgery. Identification of the most prevalent micronutrient deficiencies preoperatively, enables clinicians to limit laboratory measurements in a resource constrained setting. The results suggest that the preoperative assessment of individuals scheduled for metabolic surgery should include, at minimum, the measurement of 25(OH)D, iron studies and folate.

While initial thinking framed obesity as a disease of the developed world, evidence indicates that the developing world is equally, if not more, prone to escalating rates of obesity (29, 30). An extensive systematic review and meta-analysis conducted in 199 countries from 1980–2008 showed that the average rate of BMI increase per decade is 0.4 kg/m^2^ in men and 0.5 kg/m^2^ in women (29). The meta-analysis revealed concerning statistics specifically for South Africa, noting an increase in BMI at a rate of 2.9 kg/m^2^ per decade for males and 1.6 kg/m^2^ per decade for females during 2000–2008. Longitudinal data derived from other South African studies support these findings and corroborate an upward trend in obesity (31). In developing countries, the obesity problem is compounded by urbanization, a change in diet to low-cost, easily accessible, high sugar, carbohydrate-dense, and nutrient-deficient diets, allowing for little variety (8). Obese individuals from lower-income settings or areas where food insecurity prevails, are expected to have more profound micronutrient deficiencies. Although this study is the largest to date to investigate baseline micronutrient deficiencies before metabolic surgery in an African population, the limited number of deficiencies per income category did not allow us to perform reliable statistical analysis to assess the interplay between socio-economic and nutritional status.

Sun exposure and the presence of melanin play a significant role in the metabolism of 25(OH)D. Higher melanin concentrations have advantages for those who live in areas of intense sunlight exposure by reducing the harmful effects of ultraviolet light on the skin; however, it does reduce the efficacy of 25(OH)D metabolism. South Africa has a heterogeneous population with marked variation in skin concentration of melanin. Skin pigmentation and concentration of melanin is known to influence activation of vitamin D in the skin and varies amongst the different ethnic groups in South Africa. Globally, people of African descent with higher skin melanin content are known to be predisposed to vitamin D deficiency and lower circulating 25(OH)D levels. 25(OH)D levels were thus assessed at baseline as noted and compared amongst the different ethnic groups included in the study cohort.

A recent systematic review and meta-analysis describing 25(OH)D deficiency in Africa, with high levels of sunlight, noted a pooled prevalence of low 25(OH)D status of 18.46% (32). Further, mean serum 25(OH)D levels were lower in South Africa compared to the rest of sub-Saharan Africa (30). This was attributed to lifestyle factors and increasing urbanization (32). The optimal serum 25(OH)D for skeletal health is controversial, and concentrations for extra skeletal health have not been established. The Institute of Medicine (IOM) favors maintaining the serum 25(OH)D between 50 and 100 nmol/L for bone health (33). The US National Osteoporosis Foundation, the International Osteoporosis Foundation [IOF] and the American Geriatric Society suggest that a minimum level of 75 nmol/L is necessary to minimize fracture risk (34). Musculoskeletal health and fracture risk is a concern in people who undergo metabolic surgery, and obesity is a predisposing factor for 25(OH)D deficiency, hence the rationale for adopting the higher threshold of 72.5 nmol/L in this study to denote sufficiency and to regard a circulating level of 25(OH)D ≤ 50nmol/L as deficient.

The prevalence of 25(OH)D deficiency was high (57%) in the relatively young cohort of these study participants (mean age 45 years; [37-51 years]). The highest percentage of 25(OH)D deficiency was documented in those of mixed ancestry/Asian decent (51/65; 78%) and in black Africans (4/6; 67%), a finding that supports the notion that melanin concentration does play a role in the skin activation of 25(OH)D. It is, however, noteworthy that the prevalence of 25(OH)D deficiency in the study participants was significantly higher than the figures reported for the general population in the region thereby implicating excess body weight, irrespective of skin melanin content, as a contributor to 25(OH)D deficiency (32).

The association between 25(OH)D and obesity has not been fully established, and various theories have been postulated (35). 25(OH)D metabolism hinges on sun exposure which may be reduced in obese individuals, who tend to partake in less outdoor physical activities (18, 36). Furthermore, 25(OH)D may be sequestered in the adipose tissue of obese persons and, as such, has a reduced bioavailability (36). While the pathophysiology of 25(OH)D deficiency in obesity remains uncertain, the important role of 25(OH)D sufficiency to ensure optimal skeletal health are widely accepted. This study documented an inverse correlation between PTH and 25(OH)D. PTH secretion is expected to increase with a decrease in bioavailable 25(OH)D and a negative calcium balance. Chronic exposure of the skeleton to elevated circulating PTH is known to contribute to bone loss and decreased skeletal integrity. The findings argue for the critical interpretation of PTH to inform the routine monitoring and appropriate supplementation of 25(OH)D in obese patients, particularly those scheduled for metabolic surgery.

Most of the study participants (136/154) were women with a median age of 45 years (37–51) and the majority were either of mixed/Asian (65/154; 42%) or European (83/154; 54%) descent. Iron deficiency was noted in near half (46%) of the women in this study, with iron deficiency anemia present in 12%. A study by Phatlhane et al. in otherwise healthy non-pregnant South African adults (median age 30 years) documented iron deficiency in a concerning 56,6% of their female participants and iron deficiency anemia in 9.8% (37); findings in keeping with those described here. Diagnostic criteria employed to define iron deficiency and iron deficiency anemia were similar in this study and Phatlhane et al. (37). They attributed the presence of iron deficiency in their cohort to menstrual blood loss and limited intake of iron rich foods. In another local study, the association between obesity and iron deficiency in women aged 25-49 years in rural areas in the Free State Province of South Africa was explored. Iron-deficiency was noted in a far lower percentage of their participants (4.1%) (38). Studies from elsewhere in the world looking at micronutrient status in the morbidly obese and prior to bariatric surgery document iron deficiency in a comparatively lower percentage of their cohorts (<10%) (18, 19, 39). Detailed dietary assessment and interrogation of menstrual patterns was not performed in this study making it impossible to define causality of these findings or explain the discrepancy compared to other published data. The high prevalence of iron deficiency in the South African setting prior to metabolic surgery is important and noteworthy and should prompt assessment of iron status prior to performing metabolic surgery.

25(OH)D, folate, and iron deficiencies were more prevalent in participants with a BMI ≥45 kg/m^2,^ compared to those with a BMI <40kg/m^2^, a finding also noted in other published studies (16). In fact, folate deficiency was exclusively seen in participants in the obesity class III or above. Interpretation of this data must, however, be done cautiously as only 11 study participants, representative of a minority of the cohort (7%) had a BMI <40 kg/m^2^. Folate deficiency was observed in 18% of research participants, comparatively lower than other African studies, where folate deficiency was noted in 54% obese and non-obese women in Ghana (39). The Ghanaian study (40) notes that folate deficiency may be particularly high in West Africa where folate-deficient diets are consumed. In the same way, Modjadji et al. (41) demonstrated low folate levels in 28% of non-pregnant women of childbearing age in Limpopo, South Africa. This study was performed 4 years after the introduction of mandatory food fortification with folic acid in South Africa. Additionally, low folate levels have been linked to poor socio-economic circumstances (SES), but limited numbers precluded an evaluation of the association between folate and SES in this study (41).

We did not quantify nutrient (including folate) intake in our study, but the South African National Food Consumption Survey- Fortification Baseline (NFCS-FB) in women of reproductive age, reassuringly indicated low rates of folate deficiency nationally (42). Interestingly at the time, 60% did not look for the food fortification logo on maize, bread, or flour products (42). Provincially, women and children in the Western Cape, Northern Cape, the Free State and Eastern Cape had significantly lower mean serum and red blood cell folate compared to other provinces (42). Still, the mean folate levels in the Western Cape where 58% of women were obese or overweight was sufficient (42). It is well established that overweight and obese individuals have lower serum folate concentrations than normal-weight individuals (43). Female folate deficiency is still noted during pregnancy and lactation despite food fortification due to increased metabolic needs (40, 44). Globally the proposed contributing factors to folate deficiency in obese and overweight individuals include inflammation, insulin resistance and dysbiosis in the microbiome (43). Additional considerations are increased urinary excretion, dilution of blood volume, and impaired folate absorption by the intestinal epithelium (45). We could not identify comparable South African cohorts to juxtapose our findings scientifically and did not evaluate the influence of these factors in our study.

Clinicians should maintain a high index of suspicion and a low threshold to test for micronutrient deficiencies in obese patients, especially those with a BMI ≥40 kg/m^2^, irrespective of whether they are scheduled for metabolic surgery or not.

Evaluation of glycemic status was limited to determination of a fasting blood glucose and HbA1c and was available in 152 of study participants. Abnormal glucose homeostasis was present in a concerning number of participants (102/152; 67%). Of those 33/102 (32%) had newly diagnosed prediabetes and 13/102 (13%) unknown T2D thus representing 30% of this study cohort. Within obesity category III, 22% and 40% of women had prediabetes and T2D respectively, metabolic abnormalities not considered or required as inclusion criteria in this subgroup of the cohort. Optimal glycemic control was achieved in less than half of participants known with diabetes mellitus prior to study entry (42.8%). The high prevalence of undiagnosed prediabetes and T2D and the suboptimal control of known diabetes at baseline in subjects scheduled for bariatric surgery is noteworthy and must be addressed in clinical practice. It illustrates the significant number of unmet needs for T2D in sub-Saharan Africa (46). Evidence from the South African National Health and Nutrition Examination Survey (SANHANES-1 (2011–2012) indicated in people with T2D, 45.4% are unscreened and overall, that 80.6% of the T2D population had an unmet need for care (47). Obesity is one of the strongest risk factors for T2D and to limit the impact of this epidemic, urgent multilevel prevention and management actions are required.

This study has limitations. All participants were recruited from an existing pool of patients referred for metabolic surgery at a single tertiary hospital and extrapolation of the findings may thus not be applicable to the broader population. Findings in this study remain valuable as an initial step to uncover the baseline prevalence of micronutrient deficiencies in obese South Africans scheduled for metabolic surgery. With the projected increased prevalence of obesity on the African continent, the results caution for the judicial monitoring of patients considered for metabolic surgery as treatment method. Based on the data, it is recommended that a routine assessment of micronutrient status is performed; that as a minimum include the measurement of 25(OH)D, iron status and folate. Screening for T2D with an oral glucose tolerance test ± HbA1c determination and optimization of glycemic control in those known with T2D should be standard of care in obese individuals, including those scheduled for metabolic surgery.

## 5 Conclusion

In conclusion, the study documented micronutrient deficiencies and a concerning prevalence of abnormal glucose status in obese individuals scheduled for metabolic surgery. The appropriate detection and management of these nutritional deficiencies need to be moderated by the judicial use of resources and should include, at a minimum, the measurement of 25(OH)D and folate level and evaluation of iron status. Future efforts should seek to collate patient data on a national scale, to provide a more holistic, longitudinal picture of the relationship between obesity, metabolic surgery, and micronutrient status in the developing world, which may optimize evidence-based care.

## Data availability statement

The original contributions presented in the study are included in the article/**Supplementary Material**. Further inquiries can be directed to the corresponding authors.

## Ethics statement

The study protocol was approved by Stellenbosch University’s Health Research Ethics Committee (S18/01/003) and conducted according to the 1964 Helsinki Declaration principles. All participants provided written informed consent. The patients/participants provided their written informed consent to participate in this study.

## Author contributions

All authors have met the requirements for authorship. All authors contributed to the article and approved the submitted version.

## Supplementary material

Supplementary Table 1 Baseline characteristics and stratification categories. The WHO classification of obesity was done according to BMI (kg/m^2^): I – obese (30-34.9 kg/m^2^); II – severely obese (35-39.9 kg/m^2^); III – morbidly obese (>40 kg/m^2^). Annual Income bracket is based on the Uniform Patient Fee Schedule’s (UPFS) classification code. H0: Low – receiving grant or pension; H1: <R70 000/person or <R100 000/family; H2: R70 000- R100 000/person or R100 000-R350 000/family; H3: >R250 000/person or >R350 000/family; P: Private patients – not subsidized.
