# Supplementary material for: Nutritional deficiency in South African adults scheduled for bariatric surgery
Source: Front Endocrinol (Lausanne). 2023 May 12;14:1120531. doi: 10.3389/fendo.2023.1120531 (PMC10246427; doi:10.3389/fendo.2023.1120531)
Supplement: Supplementary Table 1 — Baseline characteristics and stratification categories. The WHO classification of obesity was done according to BMI (kg/m2): I – obese (30-34.9 kg/m2); II – severely obese (35-39.9 kg/m2); III – morbidly obese (>40 kg/m2). Annual Income bracket is based on the Uniform Patient Fee Schedule’s (UPFS) classification code. H0: Low – receiving grant or pension; H1: <R70 000/person or R250 000/person or >R350 000/family; P: Private patients – not subsidized. [file Table_1.docx]

**Supplementary Table S1. Baseline characteristics and stratification categories.**

|  | **Total study population**  (N=154) | **BMI<40kg/m^2^**  (N=11) | **BMI≥40kg/m^2^**  (N=143) | **No diabetes** (N=54) | **Pre-diabetes**  (N=34) | **Type 2 diabetes**  (N=64) |
| --- | --- | --- | --- | --- | --- | --- |
| **Baseline characteristics** *Median (IQR)* | | | | | | |
| Age (years) | 45 (37-51) | 51 (46-56) | 44 (37-50) | 41 (34-49) | 45 (40-51) | 46 (39-52) |
| Height (cm) | 163 (158-169) | 168 (161-177) | 162 (158-169) | 164 (160-168) | 163 (157-170) | 161 (158-169) |
| Weight (kg) | 135 (117-154) | 103 (97-113) | 136 (120-155) | 132 (119-151) | 133 (117-161) | 140 (116-155) |
| BMI (kg/m^2^) | 50.4 (44.6-56.5) |  |  | 48.9 (44.1-54.6) | 52.5 (44.1-56.8) | 50.3 (45.2-58.1) |
| HbA1c (%) | 5.8 (5.4-7.1) | 5.9 (5.4-7.5) | 5.8 (5.4-7) | 5.4 (5.2-5.5) | 5.9 (5.8-6.0) | 7.4 (6.6-8.8) |
| **Ethnicity/Ancestry** *(n) (%)* | | | | | | |
| Mixed or Asian | 65 (42%) | 2 (18%) | 63 (44%) | 16 (30%) | 19 (56%) | 30 (47%) |
| European | 83 (54%) | 8 (72%) | 75 (52%) | 38 (70%) | 14 (41%) | 29 (45%) |
| Black African | 6 (4%) | 1 (<1%) | 5 (3%) | 0 | 1 (3%) | 5 (8%) |
| **Gender** *(n) (%)* | | | | | | |
| Male | 18 (12%) | 3 (27%) | 15 (10%) | 4 (7%) | 3 (9%) | 11 (17%) |
| Female | 136 (88%) | 8 (72%) | 128 (90%) | 50 (93%) | 31 (91%) | 53 (83%) |
| **Obesity Classification** *(n) (%)* | | | | | | |
| Category I | 1 (<1%) |  |  | 0 | 0 | 1 (2%) |
| Category II | 10 (6,5%) |  |  | 3 (6%) | 2 (6%) | 6 (9%) |
| Category III | 143 (93%) |  |  | 51 (94%) | 32 (94%) | 57 (89%) |
| **Annual Income Bracket** *(n) (%)* | | | | | | |
| H0 | 9 (6%) | 0 | 9 (6%) | 4 (7%) | 0 | 5 (8%) |
| H1-2 | 88 (57%) | 5 (45.5%) | 83 (58%) | 30 (56%) | 19 (56%) | 38 (59%) |
| H3 | 6 (4%) | 1 (9%) | 5 (3.5%) | 3 (6%) | 3 (9%) | 0 |
| P | 51 (33%) | 5 (45.5%) | 46 (32%) | 17 (31%) | 12 (35%) | 21 (33%) |
